# The oncogenic fusion protein CBFB-SMMHC downregulates CD48 to evade NK cell recognition

**DOI:** 10.1038/s41408-018-0082-7

**Published:** 2018-05-24

**Authors:** Shira Kahlon, Dorin Shreibman, Tamar Unger, Dina Ben-Yehuda, Shlomo Elias

**Affiliations:** 1 The Lautenberg Center for General and Tumor Immunology, Department of Immunology and Cancer Research, Institute for Medical Research Israel Canada (IMRIC), Hebrew University-Hadassah Medical School, Jerusalem, Israel; 2 Department of Hematology, Hadassah – Hebrew University Medical Center, Jerusalem, Israel; 3 Center of Structural Proteomics, Weizmann Institute of Science, Rehovot, Israel

Chromosomal translocations are often found in acute leukemia and frequently result in the generation of fusion proteins with oncogenic properties^1^. We recently studied the immune evasion properties of PML-RARA and AML1-ETO, two common oncogenic fusion proteins in acute myeloid leukemia (AML). We found that both of these fusion proteins downregulate the expression of CD48, a ligand of the NK cell-activating receptor 2B4, thus leading to impaired NK cell cytotoxicity^2^. However, it remained unclear whether other leukemic fusion proteins can manipulate NK cell ligands. To explore this issue, here we tested the effects of several leukemic fusion proteins on the expression of NK cell ligands.

To examine whether NK cell ligands are downregulated by oncogenic fusion proteins other than PML-RARA and AML1-ETO^2^, we cloned several oncogenic fusion proteins associated with acute leukemia into lentiviral vectors. The oncogenic fusion proteins we examined were MLL-AF4, NUP98-HOXA9, DEK-NUP214, and CBFB-SMMHC. MLL-AF4 is associated with acute lymphoblastic lymphoma, whereas the other three fusion proteins are associated with AML. We expressed these fusion proteins in U937 cells (since this is the only cell line that could be transduced and this cell line is commonly used to express leukemic fusion proteins^3,4^). We confirmed the expression of each of these fusion proteins in U937 cells by qPCR (data not shown). Expression of MLL-AF4, NUP98-HOXA9, and DEK-NUP214 did not affect the level of several NK cell ligands including the NKG2D ligands MICA, MICB, ULBP1, ULBP2, and ULBP3 (Supplementary Figure S1). The expression of B7-H6, MHC class I (Supplementary Figure S1), and CD48 (Fig. 1a) was also not affected. By contrast, the expression of the fusion protein CBFB-SMMHC led to a nearly complete abolishment of CD48 expression while not affecting the other NK cell ligands (Fig. 1b).

**Fig. 1:**
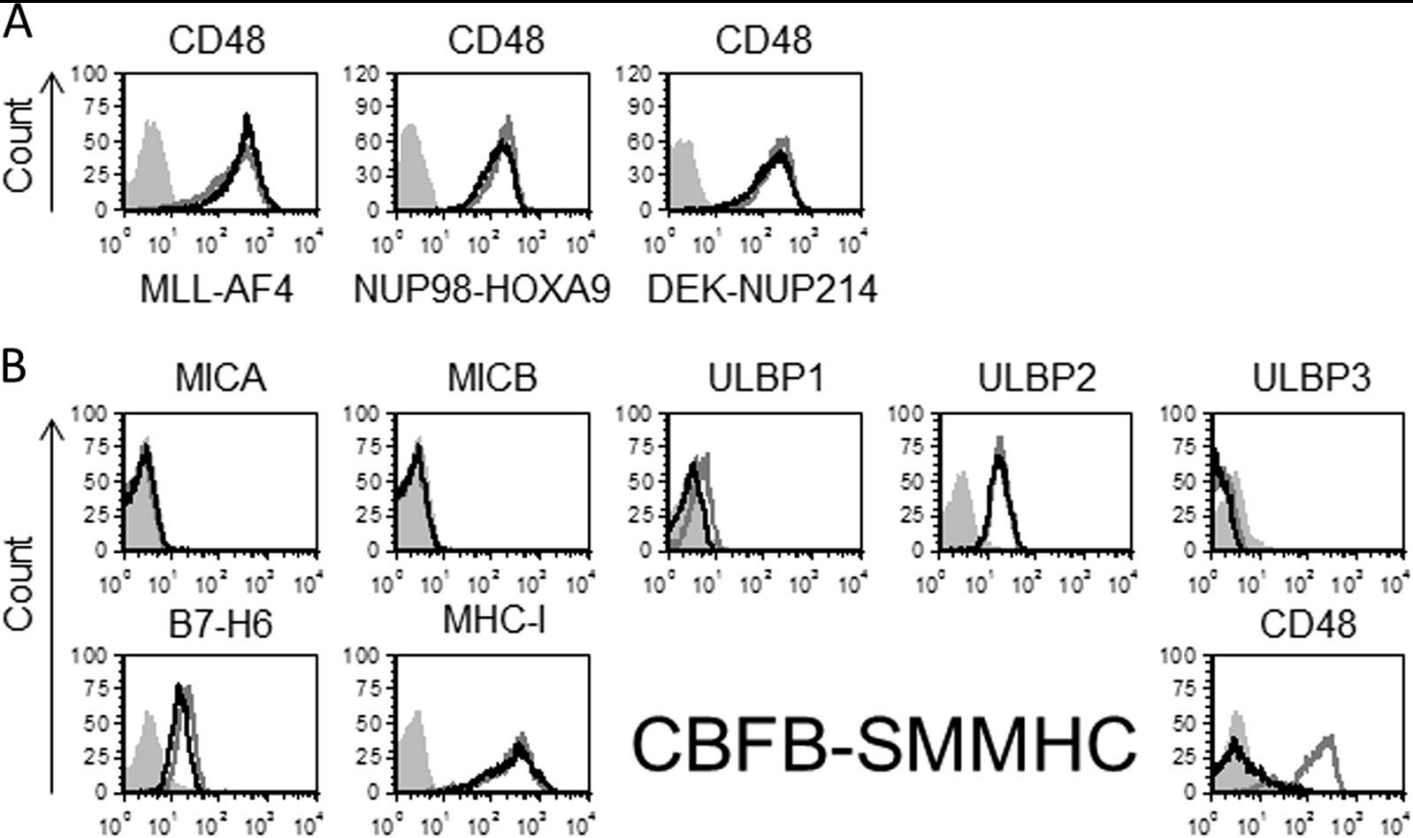
The effect of various fusion proteins on the expression of NK cell ligands. **a**, **b** Flow cytometry analysis of various NK cell ligands expressed by U937 cells transduced with the AML fusion proteins MLL-AF4, NUP98-HOXA9, or DEK-NUP214 (**a**) and CBFB-SMMHC (**b**; black lines), compared to U937 cells transduced with an empty vector (gray lines). Gray-shaded histograms, background staining with an isotype-matched control antibody. The figure shows one representative experiment out of at least two performed

Next, we tested the functional significance of the downregulation of CD48 by CBFB-SMMHC by performing cytotoxicity assays with NK cells. We first used the NK cell line YTS eco since the cytotoxicity of these cells is mainly dependent on the 2B4–CD48 interaction^2^. We found that cells that express CBFB-SMMHC were killed significantly less than the control cells (Fig. 2a). To verify that the killing was indeed mediated by CD48, we blocked CD48 on the target cells and observed almost no killing of any of the targets (Fig. 2b).

**Fig. 2:**
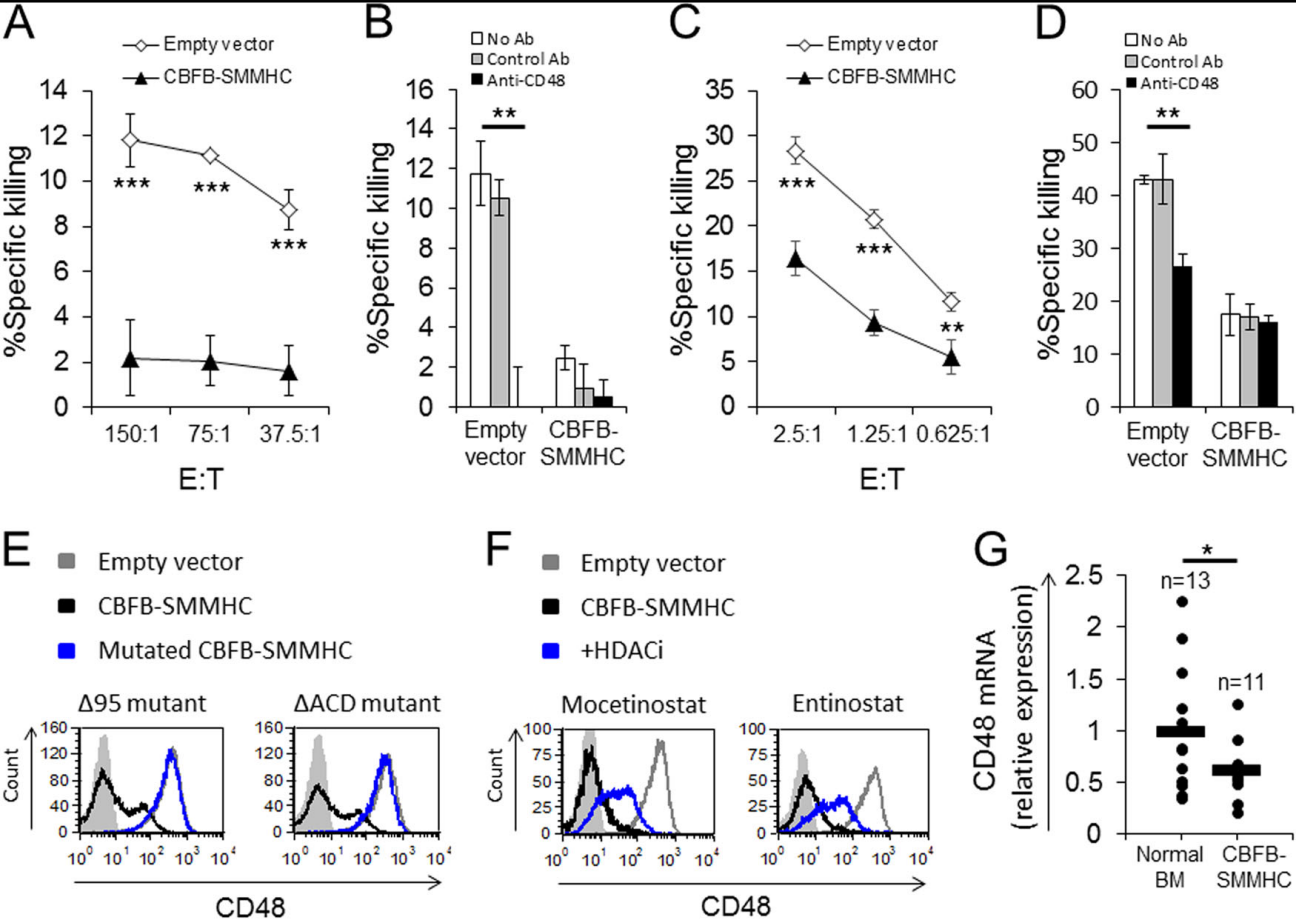
Functional significance and mechanism governing the downregulation of CD48 by CBFB-SMMHC. **a–d** Cytotoxicity assays. ^35^S-labeled U937 cells expressing CBFB-SMMHC or an empty vector were pre-incubated with or without anti-CD48 or control mAb and then incubated with YTS eco cells (**a**, **b**) or with primary NK cells (**c**, **d**). The effector to target (E:T) ratios were 50:1 (**b**), 2.5:1 (**d**), or indicated on the *x*-axis (**a**, **c**). Error bars represent the standard deviation of triplicates. One representative experiment is shown out of three performed. (**e**) Flow cytometry analysis of CD48 level in cells expressing two deletion mutants of CBFB-SMMHC (both in blue histograms): Δ95 (left) and ΔACD (right) in parallel to staining of cells expressing the WT CBFB-SMMHC protein (black histograms) or an empty vector (gray histograms). The analysis was performed with gating on GFP+ cells. Gray-shaded histogram, background staining with an isotype-matched control antibody. One representative experiment is shown out of two performed. **f** Flow cytometry analysis of CD48 expression in U937 cells transduced with CBFB-SMMHC and treated with HDAC inhibitors (HDACi). Cells transduced with an empty vector were treated with a solvent control (gray lines); cells transduced with CBFB-SMMHC were treated with a solvent control (black lines) or with two different HDACi (blue lines): mocetinostat and entinostat. Gray-shaded histogram, background staining with an isotype-matched control antibody. The figure shows one representative experiment out of two performed. **g** Expression of CD48 in normal bone marrow samples (left) and in bone marrow samples of AML patients expressing CBFB-SMMHC (right) as determined by qRT-PCR. Black line represents the mean value. In all figure panels, *P < 0.05, **P < 0.01, ***P < 0.001, Student’s *t-*test

We also tested the killing of CBFB-SMMHC-expressing cells by IL-2-activated primary bulk NK cells and found that cells expressing the CBFB-SMMHC fusion protein were killed significantly less than cells that expressed an empty vector (Fig. 2c). The blocking of CD48 on the target cells significantly reduced the killing of all cells to a similar extent (Fig. 2d). Hence, the downregulation of CD48 by CBFB-SMMHC is functional and leads to reduced NK cell-mediated killing.

To test the mechanism by which CBFB-SMMHC influences CD48 expression, we first tested the mRNA levels of CD48 by qPCR after overexpression of this fusion protein. We found that overexpression of CBFB-SMMHC reduced the mRNA levels of CD48 (Supplementary Figure S2). Next, we tested whether the effect of the CBFB-SMMHC protein on the expression of CD48 depends on recruitment of histone deacetlyase (HDAC). We generated two deletion mutants of CBFB-SMMHC, which have been shown to affect the activity of this protein by abolishing binding to HDAC^5,6^: CBFB-SMMHC Δ95 (which lacks 95 amino acids at the C terminus) and CBFB-SMMHC ΔACD (which lacks amino acids 514–542). Both of these mutated CBFB-SMMHC proteins were unable to downregulate CD48 expression (Fig. 2e). We also examined the possibility that treatment with HDAC inhibitors (HDACi) could reverse the downregulation of CD48 by CBFB-SMMHC. Treatment with two specific class I HDACi, mocetinostat and entinostat, upregulated the expression of CD48 in cells that expressed CBFB-SMMHC (Fig. 2f).

To test whether our findings are relevant to human AML patients, we analyzed the expression of CD48 in AML patients expressing CBFB-SMMHC. We collected bone marrow aspirations of AML patients who express CBFB-SMMHC as well as normal bone marrow samples. The relative expression of CD48 in these samples was determined by qRT-PCR using specific primers for CD48. This analysis indicated that AML patients who express CBFB-SMMHC have lower expression of CD48 as compared to normal bone marrow samples (Fig. 2g).

CBFB-SMMHC is a common fusion protein in AML that is a result of inv(16) or t(16;16), which lead to juxtaposition of the CBFB and MYH11 genes^7^. This fusion protein is related to the AML French-American-British (FAB) subtype M4Eo^8^. In line with previous reports^5,6^, our findings support the role of HDAC in the oncogenicity of this fusion protein since deletion of the HDAC-binding site abolished the effect on CD48 and treatment with HDACi reversed the expression of CD48 in cells that express this fusion protein.

Today, it is widely recognized that NK cells play a significant role in eliminating AML cells (see, for example, ref.^9^). We have reported that two common oncogenic fusion proteins in AML, PML-RARA and AML1-ETO, downregulate the expression of CD48^2^. AML with expression of AML1-ETO or CBFB-SMMHC is clinically classified as core-binding factor (CBF)-AML since both of these fusion proteins involve members of the CBF (RUNX1 and CBFB, respectively)^10^. Although AML1-ETO and CBFB-SMMHC share several similarities, they differ clinically and mechanistically, for example, in their genomic binding regions ^11,12^.

The three fusion proteins we found to downregulate the expression of CD48 (PML-RARA, AML1-ETO, and CBFB-SMMHC) are associated with better prognosis in AML^13^. However, ~30% of CBF-AML patients eventually relapse^14^. On the basis of our findings we suggest that downregulation of CD48 by these fusion proteins, which leads to NK cell immune evasion, contributes to the persistence of a residual disease in these subtypes of leukemia and eventually to a clinical relapse. The other fusion proteins we tested are associated with more aggressive types of acute leukemia^13,15^ and, therefore, are probably less dependent on NK cell immune evasion. Thus, NK cell-based therapies (i.e., NK cell infusion) or class I HDACi may be potential adjunctive therapies for CBF-AML or APL.

## Electronic supplementary material

Supplemental figures

Supplemental methods
