# Supplementary material for: The oncogenic fusion protein CBFB-SMMHC downregulates CD48 to evade NK cell recognition
Source: Blood Cancer J. 2018 May 24;8(5):48. doi: 10.1038/s41408-018-0082-7 (PMC5968028; doi:10.1038/s41408-018-0082-7)
Supplement: Supplementary file 1 — Supplemental figures [file 41408_2018_82_MOESM1_ESM.pdf]

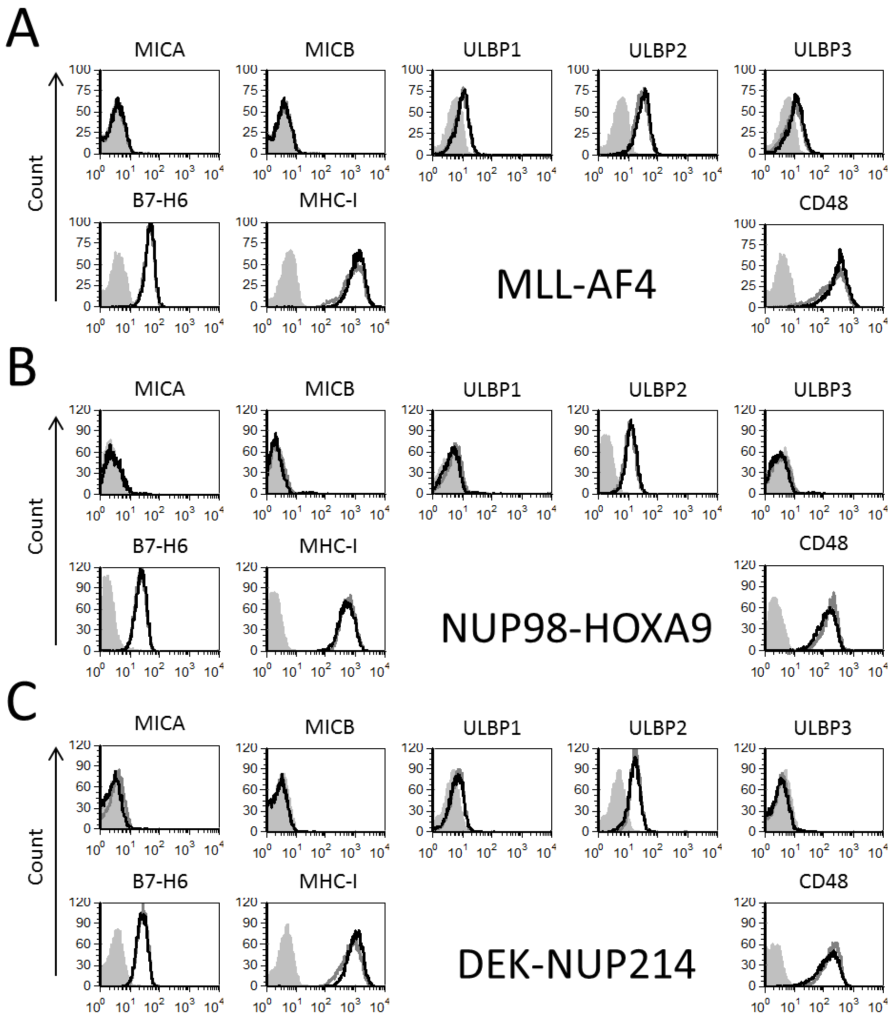

Figure S1

Figure S1. The effect of various fusion proteins on the expression of NK cell ligands

(A,B,C) Flow cytometry analysis of various NK cell ligands expressed by U937 cells transduced with the AML fusion proteins MLL-AF4 (A), NUP98-HOXA9 (B) or DEK-NUP214 (C) (black lines), compared to U937 cells transduced with an empty vector (gray lines). Gray shaded histograms, background staining with an isotype-matched control antibody. The figure shows one representative experiment out of two performed. The histograms from three or two separate experiments were merged in (B) and (C) respectively.

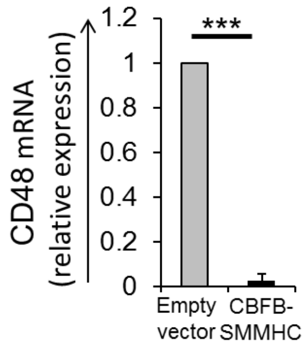

Figure S2

Figure S2. The effect of CBFB-SMMHC on the mRNA level of CD48.

qRT-PCR analysis of the expression of CD48 in U937 cells transduced with an empty vector compared to U937 transduced with CBFB-SMMHC. The results were normalized to the expression of HPRT. The relative copy number of CD48 in cells expressing the empty vector was defined as 1. Error bars represent the standard deviation of the means of three experiments (each in triplicate).
