# Supplementary material for: The oncogenic fusion protein CBFB-SMMHC downregulates CD48 to evade NK cell recognition
Source: Blood Cancer J. 2018 May 24;8(5):48. doi: 10.1038/s41408-018-0082-7 (PMC5968028; doi:10.1038/s41408-018-0082-7)
Supplement: Supplementary file 2 — Supplemental methods [file 41408_2018_82_MOESM2_ESM.pdf]

Supplementary methods

Cloning, production of lentiviral constructs and viral transduction

The fusion proteins were cloned into lentiviral vectors using a restriction free (RF) methodology.[1] DEK-NUP214 and NUP98-HOXA9 (original plasmids, kindly provided by Urban Gullberg of Lund University) were cloned into the pRRL-EF1a-PGK-NEO vector, and CBFB-MYH11 (which encodes the CBFB-SMMHC protein, original plasmid kindly provided by Paul Liu of the National Human Genome Research Institute) was cloned into the pHAGE-DsRED(−)-eGFP(+) vector. In addition, to express MLL-AF4 we used the lentiviral vector pRRL-EF1a-MLL-AF4-PGK-NEO (kindly obtained from Pablo Menéndez of the University of Barcelona). We also generated two deletion mutants of CBFB-SMMHC[2,3] which were cloned to the pHAGE-DsRED(−)-eGFP(+) vector as the WT protein: CBFB-SMMHC which lacks 95 amino acids at the C terminus (CBFB-SMMHC Δ95) and CBFB-SMMHC with the omission of amino acids 514-542 (CBFB-SMMHC ΔACD).

Lentiviral virions were produced by transient three-plasmid transfection of 293T cells as previously described.[4] These viruses were used to transduce U937 cells. Infected U937 cells were selected by G418 (in the case of the pRRL-EF1a-PGK-NEO vector) or by GFP (in the case of the pHAGE-DsRED(−)-eGFP(+) vector).

Cells and Antibodies

The cell lines used in this study were U937 and the human NK cell line YTS eco. Primary NK cells were isolated from the peripheral blood of healthy human volunteers as previously described.[5] NK cells were stained with PE anti-CD56 (cat. 318306, BioLegend) and anti-CD3-Allophycocyanin (cat. 300312, BioLegend) to confirm NK purity after isolation and following activation.

We analyzed the expression of NK cell ligands on U937 cells by flow cytometry after blocking with 20% human serum since these cells express Fc receptors.[6]

To stain NK cell ligands we used the following antibodies: anti-CD48 (sc-8397, Santa Cruz Biotechnology), anti-MICA (MAB 1300, R&D), anti-MICB (MAB1599, R&D), anti-ULBP1 (MAB1380, R&D), anti-ULBP2 (MAB1298, R&D), anti-ULBP3 (MAB1517, R&D), anti-B7-H6 (Human B7-H6 Mab7144), and the anti-MHC class I W6/32 (produced from a hybridoma).

We used the following isotype controls: mouse IgG1 (cat. 400102, BioLegend or cat. 14-4714-85, mbioscience); mouse IgG2a (cat. 400202, BioLegend); mouse IgG2b (cat. 400302, BioLegend).

For secondary antibody staining we used the antibody Alexa Fluor 647-conjugated AffiniPure F(ab')2 Fragment Goat anti-mouse (115-606-062, Jackson ImmunoResearch Laboratories).

Drugs

We used the following histone deacetylase inhibitors (HDACi): mocetinostat (MGCD0103, cat. num. S1122, Selleck Chemicals) dissolved in dimethyl sulfoxide, at a final concentration of 1µM;[7] entinostat (MS-275, cat. num. 27011, BPS Bioscience) dissolved in dimethyl sulfoxide, at a final concentration of 1µM.[7] Both HDAC inhibitors were used for 18-24 hours.

Cytotoxicity assay

The *in-vitro* cytotoxic activity of NK cells against various targets was assessed in 5-hour ^{35}S-release assays as described previously.[5] We used the anti-CD48 blocking

antibody (sc-8397, Santa Cruz Biotechnology) and the control antibody anti-HA (12CA5). The final concentration of the blocking antibodies was 0.5 μg/well.

Quantitative Real-Time PCR (qRT-PCR)

For mRNA quantification in cell lines, total RNA was isolated from cells using the Quick-RNA MiniPrep kit (Zymo research). For mRNA quantification of human samples, total RNA of bone marrow aspirates was extracted with TRI-reagent (MRC). The collection of patient samples was approved by the Institutional Helsinki Committee of Hadassah Medical Center. AML patients with inv(16) were identified by molecular testing or cytogenetic analysis.

RNA was reverse transcribed with Moloney murine leukemia virus reverse transcriptase (Invitrogen) and with polyT primer (Thermo Scientific). Quantitative amplification was conducted on a QuantStudio 12K Flex Real-Time PCR System (Applied Biosystems) with gene specific primers and Platinum SYBR Green qPCR Super Mix-UDG with ROX (ThermoFisher Scientific). For the qPCR of cell lines HPRT was used as a reference gene. For the qRT-PCR of human samples, we used 3 reference genes (HPRT, SDHA, UBC). In addition, for the analysis of the human samples, the CD48 level in one arbitrary sample was defined as one (1) and the CD48 level in the rest of samples was compared to this reference sample. Afterwards, the relative expression of CD48 in each sample was normalized to the mean relative expression of CD48 in the normal bone marrow samples (so that the mean CD48 expression of this group was one).

We used the following primers for qRT-PCR:

MLL-AF4 fwd - 5'- CAGAGCAAACAGAAAAAAGTG -3’

MLL-AF4 rev - 5’- GTTCTGGAAGGGACTGTGGA -3’

MLL-AF4 fwd - 5'- CAGGTCCAGAGCAGAGCAAAC -3’

MLL-AF4 rev - 5’- GAGCACTTGGAGGTGCAGATG -3’

NUP98-HOXA9 fwd - 5'- GGGCACCGCTTTTTCCGAGT -3'

NUP98-HOXA9 rev – 5'- TCTTGGTACAGGAGCCTTTGGG -3'

DEK-NUP214 fwd – 5'- AGCAGCACCACCAAGAAGAAT -3'

DEK-NUP214 rev – 5'- GTCTCTCGCTCTGGCACAAG -3'

CBFB-MYH11 fwd - 5'- GCAGGCAAGGTATATTTGAAGG -3'

CBFB-MYH11 rev - 5'- CTCTTCTCCTCATTCTGCTCGT-3'

CBFB-MYH11 fwd - 5'-AAGACTGGATGGTATGGGCTGT -3'

CBFB-MYH11 rev - 5'-CAGGGCCCGCTTGGA-3'

CD48 fwd – 5'-GGAATTGCTACTGCTGCCTC-3'

CD48 rev – 5'-AGTTCTCAGGCAGGCTCTCA-3'

HPRT fwd – 5'-TGACACTGGCAAAACAATGCA-3'

HPRT rev – 5'-GGTCCTTTTCACCAGCAAGCT-3'

SDHA fwd – 5'- TGCAGAACCTGATGCTGTGT-3'

SDHA rev – 5'- CCAGAGTGACCTTCCCAGTG-3'

UBC fwd – 5'-ATTTGGGTCGCGGTTCTTG-3'

UBC rev – 5'-TGCCTTGACATTCTCGATGGT-3'

References

1. Unger T, Jacobovitch Y, Dantes A, Bernheim R, Peleg Y. Applications of the Restriction Free (RF) cloning procedure for molecular manipulations and protein expression. *J Struct Biol*. 2010;172(1):34-44.
2. Qi J, Singh S, Hua WK, et al. HDAC8 Inhibition Specifically Targets Inv(16) Acute Myeloid Leukemic Stem Cells by Restoring p53 Acetylation. *Cell Stem Cell*. 2015;17(5):597-610.
3. Durst KL, Lutterbach B, Kummalue T, Friedman AD, Hiebert SW. The inv(16) fusion protein associates with corepressors via a smooth muscle myosin heavy-chain domain. *Mol Cell Biol*. 2003;23(2):607-619.
4. Elias S, Yamin R, Golomb L, et al. Immune evasion by oncogenic proteins of acute myeloid leukemia. *Blood*. 2014;123(10):1535-1543.

5. Yamin R, Lecker LS, Weisblum Y, et al. HCMV vCXCL1 Binds Several Chemokine Receptors and Preferentially Attracts Neutrophils over NK Cells by Interacting with CXCR2. *Cell Rep*. 2016;15(7):1542-1553.
6. Looney RJ, Abraham GN, Anderson CL. Human monocytes and U937 cells bear two distinct Fc receptors for IgG. *J Immunol*. 1986;136(5):1641-1647.
7. Duque-Afonso J, Yalcin A, Berg T, Abdelkarim M, Heidenreich O, Lubbert M. The HDAC class I-specific inhibitor entinostat (MS-275) effectively relieves epigenetic silencing of the LAT2 gene mediated by AML1/ETO. *Oncogene*. 2011;30(27):3062-3072.
